# Overexpression of the Mg-chelatase H subunit in guard cells confers drought tolerance via promotion of stomatal closure in *Arabidopsis thaliana*

**DOI:** 10.3389/fpls.2013.00440

**Published:** 2013-10-30

**Authors:** Tomo Tsuzuki, Koji Takahashi, Masakazu Tomiyama, Shin-ichiro Inoue, Toshinori Kinoshita

**Affiliations:** ^1^Division of Biological Science, Graduate School of Science, Nagoya UniversityNagoya, Japan; ^2^Institute of Transformative Bio-Molecules (WPI-ITbM), Nagoya UniversityNagoya, Japan

**Keywords:** stomata, CHLH, ABA, drought tolerance, H^+^-ATPase, *Arabidopsis*

## Abstract

The Mg-chelatase H subunit (CHLH) has been shown to mediate chlorophyll biosynthesis, as well as plastid-to-nucleus and abscisic acid (ABA)-mediated signaling. A recent study using a novel *CHLH* mutant, *rtl1*, indicated that CHLH specifically affects ABA-induced stomatal closure, but also that CHLH did not serve as an ABA receptor in *Arabidopsis thaliana*. However, the molecular mechanism by which CHLH engages in ABA-mediated signaling in guard cells remains largely unknown. In the present study, we examined CHLH function in guard cells and explored whether *CHLH* expression might influence stomatal aperture. Incubation of *rtl1* guard cell protoplasts with ABA induced expression of the ABA-responsive genes *RAB18* and *RD29B*, as also observed in wild-type (WT) cells, indicating that *CHLH* did not affect the expression of ABA-responsive genes. Earlier, ABA was reported to inhibit blue light (BL)-mediated stomatal opening, at least in part through dephosphorylating/inhibiting guard cell H^+^-ATPase (which drives opening). Therefore, we immunohistochemically examined the phosphorylation status of guard cell H^+^-ATPase. Notably, ABA inhibition of BL-induced phosphorylation of H^+^-ATPase was impaired in *rtl1* cells, suggesting that CHLH influences not only ABA-induced stomatal closure but also inhibition of BL-mediated stomatal opening by ABA. Next, we generated *CHLH-GFP-*overexpressing plants using *CER6* promoter, which induces gene expression in the epidermis including guard cells. *CHLH*-transgenic plants exhibited a closed stomata phenotype even when brightly illuminated. Moreover, plant growth experiments conducted under water-deficient conditions showed that *CHLH* transgenic plants were more tolerant of drought than WT plants. In summary, we show that *CHLH* is involved in the regulation of stomatal aperture in response to ABA, but not in ABA-induced gene expression, and that manipulation of stomatal aperture via overexpression of *CHLH* in guard cells improves plant drought tolerance.

## Introduction

Stomatal pores, surrounded by paired guard cells and located in the epidermis of plants, regulate gas exchange in response to both environmental and endogenous signals. Stomatal opening facilitates both transpiration and CO_2_ entry (to enable photosynthesis) (Shimazaki et al., 2007). Stomatal opening is triggered by blue light (BL) via activation of the plasma membrane H^+^-ATPase of guard cells (Assmann et al., 1985; Shimazaki et al., 1986; Kinoshita and Shimazaki, 1999). BL-induced activation of H^+^-ATPase involves phosphorylation of the penultimate C-terminal threonine (Thr) of the enzyme (Kinoshita and Shimazaki, 2002; Kinoshita and Hayashi, 2011).

When water is scarce, the plant hormone abscisic acid (ABA) promotes stomatal closure to prevent water loss (Schroeder et al., 2001). ABA-induced stomatal closure is driven by an efflux of K^+^ from guard cells; the ions pass through voltage-dependent outward-rectifying K^+^ channels in the plasma membrane. Activation of K^+^ channels requires depolarization of the membrane, achieved principally by activating anion channels within the membrane (Schroeder et al., 1987; Negi et al., 2008; Vahisalu et al., 2008; Kim et al., 2010). ABA-dependent depolarization of the membrane has been suggested to be achieved in part by the inhibition of plasma membrane H^+^-ATPase action (Shimazaki et al., 1986; Goh et al., 1996; Roelfsema et al., 1998). In support of this contention, an *ost2* missense mutant that constitutively expresses a highly active form of H^+^-ATPase AHA1 did not exhibit ABA-induced stomatal closure (Merlot et al., 2007). Inhibition of H^+^-ATPase by ABA is attributable to reduced phosphorylation of the penultimate Thr of the enzyme (Zhang et al., 2004; Hayashi et al., 2011). More recently, three basic helix-loop-helix (bHLH) transcription factors termed AKSs (ABA-responsive kinase substrates) have been shown to become phosphorylated in response to ABA (Takahashi et al., 2013). Proteins in the AKS family facilitate stomatal opening by increasing transcription of genes encoding inward-rectifying K^+^ channels, and ABA suppresses the activities of these channels by triggering the phosphorylation of transcription factors in the AKS family.

Several ABA receptors have been described. These include the Mg-chelatase H subunit (CHLH) (Shen et al., 2006; Wu et al., 2009; Du et al., 2012), G-protein-coupled receptor 2 (GCR2) (Liu et al., 2007), and the G-protein-coupled receptor-type G proteins GTG1 and GTG2 (Pandey et al., 2009). However, the suggestion that CHLH and GCR2 act as ABA receptors remains controversial (McCourt and Creelman, 2008; Cutler et al., 2010). Apart from these candidates, a family of START domain proteins, termed the PYR/PYL/RCARs (pyrabactin resistace/pyrabactin resistance 1-like/regulatory component of ABA receptor) has recently been convincingly identified as cytosolic ABA receptors, and recognition of ABA by these proteins activates members of the protein kinase SRK2/SnRK2 family, including OST1, via inactivation of centrally acting negative regulators, the type 2C protein phosphatases (PP2Cs) that include ABI1 and ABI2 (Ma et al., 2009; Park et al., 2009; Kim et al., 2010; Nishimura et al., 2010). Today, an early acting ABA-signaling pathway, PYR/PYL/RCARs-PP2Cs-SRK2/SnRK2s, is considered to mediate most ABA-triggered plant responses, including the inhibition of seed germination and root growth, stomatal closure, and the expression of particular genes (Cutler et al., 2010). Notably, ABA-dependent phosphorylation of the bHLH transcription factors (the AKSs in guard cells) is also mediated by the PYR/PYL/RCARs-PP2Cs-SRK2/SnRK2s pathway (Takahashi et al., 2013).

The CHLH of chloroplasts was reported to serve as an ABA receptor, mediating ABA-dependent inhibition of seed germination and root growth, ABA-induced expression of certain genes, and ABA-dependent stomatal closure (Shen et al., 2006; Wu et al., 2009; Du et al., 2012). CHLH is a multifunctional protein involved in chlorophyll synthesis (Gibson et al., 1995; Willows et al., 1996; Huang and Li, 2009), retrograde plastid-to-nucleus signaling (Mochizuki et al., 2001), and ABA signaling. Recently, a *rapid transpiration in detached leaves 1* (*rtl1*) mutant bearing a novel missense mutation in the H subunit of Mg-chelatase (CHLH) was isolated from ethyl methanesulfonate (EMS)-treated *Arabidopsis thaliana* using screen seeking mutants altered in terms of stomatal aperture (Tsuzuki et al., 2011). Stomatal movements in the *rtl1* mutant were insensitive to ABA, but ABA continued to normally influence seed germination and root growth. Moreover, the recombinant CHLH protein did not bind to [^3^H]-labeled ABA. Therefore, the question of whether CHLH indeed functions as an ABA receptor, and mediates ABA-dependent inhibition of seed germination and root growth (Müller and Hansson, 2009; Tsuzuki et al., 2011), remains controversial. Moreover, the *rtl1* mutant exhibited ABA-induced stomatal closure in the presence of a high concentration of extracellular Ca^2+^, suggesting that *CHLH* affects Ca^2+^ signaling in guard cells (Tsuzuki et al., 2011). However, the molecular mechanism by which CHLH mediates ABA-signaling in such cells remains largely unknown.

In the present study, we further analyzed CHLH function in guard cells and tried to manipulate stomatal aperture using *CHLH*. First, we examined ABA-dependent gene expression and phosphorylation of AKSs in guard cell protoplasts (GCPs) from the *rtl1* mutant and concluded that CHLH did not affect these processes. Furthermore, we found that CHLH reduced the ABA mediated de-phosphorylation of guard-cell H^+^-ATPase. Finally, we obtained direct evidence that overexpression of CHLH in guard cells confers drought tolerance on *Arabidopsis* plants by increasing the sensitivity of stomatal guard cells to ABA.

## Materials and methods

### Plant materials and growth conditions

Plants of *A. thaliana* ecotype Columbia *gl1* (*glabrous1*) served as wild-type (WT) plants. The background ecotype of the *rtl1* mutation is *gl1* (Tsuzuki et al., 2011), L*er* (Landsberg *erecta*) is the background ecotype of *abi1-1* (Leung et al., 1997), and Col is the background ecotype of a *cch* mutant (Mochizuki et al., 2001). The *abi1-1* mutant was obtained from the Arabidopsis Biological Resource Center (Ohio State University, Columbus, OH, USA). Plants were grown in soil under a 16-h fluorescent light (50 μmol m^−2^ s^−1^)/8-h dark cycle at 24°C at a relative humidity of 55–75% for 4–6 weeks in a dedicated growth room (Kinoshita et al., 2001).

### Isolation of GCPs and ABA treatment

GCPs from 5-week-old rosette leaves (~10 g) were enzymatically prepared as described by Ueno et al. (2005). To analyze ABA-responsive gene expression, GCPs were incubated in buffer (5 mM MES-NaOH, 10 mM KCl, 0.4 M mannitol, 1 mM CaCl_2_) supplemented (or not) with 20 μM ABA in the dark for 1 h at 24°C. For protein blot analysis and detection of 14-3-3 protein, GCPs were incubated in the above buffer with or without 20 μM ABA in the dark for 10 min at 24°C.

### Quantification of mRNAs transcribed from ABA-responsive genes

Real-time quantitative polymerase chain reaction (PCR) was performed as described by Kinoshita et al. (2011) using the Power SYBR Green PCR Master Mix and the Step One Real-Time PCR system (Applied Biosystems, Foster City, CA, USA). Total RNA was isolated from GCPs using RNeasy Plant Mini Kits (Qiagen, Valencia, CA, USA), and first-strand cDNA was synthesized using Takara PrimeScript II 1st Strand cDNA Synthesis Kits (Takara, Tokyo, Japan). To specifically amplify *RAB18* (At5g66400) and *RD29B* (At5g52300) transcripts, the following primer sets were used: for *RAB18*, 5′-TGTAACGCAGTCGCATTCG-3′ and 5′-CACATCGCAGGACGTACATACAT-3′; and for *RD29B*, 5′-CGAGCAAGACCCAGAAGTTCAC-3′ and 5′-TTACCCGTTACACCACCTCTCA-3′. Relative quantification of target gene expression levels was achieved using the comparative Ct (threshold cycle) method, and expression levels were normalized to that of *TUB2* (At5g62690) transcripts (internal control) amplified using the primer pair 5′-AAACTCACTACCCCCAGCTTTG-3′ and 5′-CACCAGACATAGTAGCAGAAATCAAGT-3′. Three replicates were prepared in each experiment and the assays were repeated three times.

### Immunoblot and protein blot analysis

Immunoblotting for 14-3-3 protein and protein blot analysis of GCPs were conducted as described by Takahashi et al. (2013). Thirty microgram amounts of guard cell proteins were separated by sodium dodecyl sulfate-polyacrylamide gel electrophoresis and transferred to nitrocellulose membranes. Twenty-five microgram amounts of protein from epidermal fragments were used when immunoblotting for CHLH. Epidermal fragments, including guard cells, were obtained as described by Hayashi et al. (2011), with some modifications. Fully expanded rosette leaves were harvested from each experimental dark-adapted 4–6 week-old plant and blended for 7 s in 35 ml of Milli-Q water (Millipore, Billerica, MA, USA) using a Waring blender equipped with an MC1 mini container (Waring Commercial, Odessa, FL, USA). Epidermal fragments were collected by retention on nylon mesh (58 μm in pore diameter), rinsed in Milli-Q water, and stored in a basal buffer containing 5 mM MES-BTP (pH 6.5), 50 mM KCl, and 0.1 mM CaCl_2_. The 14-3-3 proteins and CHLH were detected using an anti-14-3-3 protein (GF14ø) antibody and an anti-CHLH antibody, respectively (Kinoshita and Shimazaki, 1999; Tsuzuki et al., 2011); both antibodies were used at 3000-fold dilutions. An anti-goat IgG antibody conjugated with horseradish peroxidase (HRP) (Bio-Rad Laboratories, Hercules, CA, USA) was used at a dilution of 1:3,000 as a secondary antibody, and incubation proceeded at room temperature for 2 h. Chemiluminescence developing upon reaction of HRP with a substrate (Pierce, Rockford, IL, USA) was detected using the Light Capture AE-2150 system (Atto, Tokyo, Japan). For protein blot analysis, each nitrocellulose membrane was incubated with 0.1 μM GST-14-3-3 for 16 h at 4°C, and next with an anti-GST antibody (GE Healthcare UK Ltd., Buckinghamshire, UK) at a dilution of 1:3000 for 2 h at room temperature. The membrane was then exposed to an anti-goat IgG secondary antibody conjugated with HRP at a dilution of 1:3000 for 2 h at room temperature and the HRP-substrate reaction detected as described above.

### Immunohistochemical detection of plasma membrane H^+^-ATPase in guard cells

Epidermal fragments were incubated for 20 min under background red light (RL; LED-R, Eyela, Tokyo, Japan) at 50 μmol m^−2^ s^−1^. BL (Stick-B-32, Eyela) at 10 μmol m^−2^ s^−1^ was applied to RL-illuminated epidermal fragments, thus being superimposed on the background RL, for 2.5 min. The phosphorylation status of epidermal guard cell H^+^-ATPase was immunohistochemically evaluated using the method of Hayashi et al. (2011). Epidermal fragments were incubated with anti-H^+^-ATPase and anti-pThr antibodies, which specifically recognize the catalytic domain and the phosphorylated threonine, respectively, of H^+^-ATPase (Hayashi et al., 2010). Both antibodies were added at a dilution of 1:1000 (w/v) in phosphate-buffered saline (PBS; 137 mM NaCl, 8.1 mM Na_2_HPO_4_, 2.68 mM KCl, 1.47 mM KH_2_PO_4_) with 3% (w/v) bovine serum albumin Fraction V (BSA; Sigma, St. Louis, MO, USA) and incubation proceeded at 37°C overnight. All samples were washed six times for 5 min each time with PBS and incubated with Alexa Fluor 488 goat anti-rabbit IgG (Invitrogen, Carlsbad, CA, USA) at a dilution of 1:500 in PBS with 3% (w/v) BSA at 37°C for 3 h in the dark. After washing a further six times for 5 min each time with PBS, each specimen was mounted on a glass slide using 50% (v/v) glycerol. Specimens were observed under a fluorescence microscope (BX50; Olympus, Tokyo, Japan) using the narrow excitation band-pass filter set BP460–480HQ BA495–540HQ (U-MGFPHQ; Olympus) to detect signal from Alexa Fluor 488. A Hg arc lamp was used as a source of excitation. Fluorescent images were collected using a CCD camera (DP72; Olympus) and processed with the aid of DP2-BSW software (Olympus). To estimate fluorescence intensities, all exposure times were identical (334.79 ms). The fluorescence intensities of stomata (>30 stomata/treatment) were determined via a zone densitometry method using CS Analyzer version 3.0 (Atto).

### Measurement of stomatal aperture

Stomatal apertures were measured as described by Inoue et al. (2008). The stomatal aperture in the abaxial epidermal tissues of rosette leaves was measured microscopically. Stomatal apertures were the mean of 25 stomata and are shown with standard deviations (SDs). The data were confirmed by blind reassessment.

### Drought tolerance test

Plants were grown in soil for 3 weeks under normal condition and then subjected to drought stress by withholding water for 18 days (Iuchi et al., 2001).

### Vector construction and transformation

*CHLH* full-length cDNA was amplified by PCR with insertion of *Sal*I sites upstream of the start codon and downstream of the stop codon using the primer pair 5′-ACGCGTCGACAAAATGGCTTCGCTTGTGTATTCTCC-3′ and 5′-ACGCGT CGACTCGATCGATCCCTTCGATCTTG-3′. The resulting PCR product was digested with *Sal*I and subcloned into plasmid pUC18-*CaMV35S::GFP* (S65T) in a manner causing the 3′-end of the *CHLH* coding sequence to be fused in-frame to the 5′-end of *GFP*. *CHLH-GFP* was amplified by PCR with insertion of *Xba*I sites upstream of the *CHLH* start codon and downstream of the *GFP* stop codon using the primer pair 5′-GCCTCTAGAAAAATGGCTTCGCTTGTGTATTCTCCAT-3′ and 5′-GCCTCTAGATTACTTGTACAGCTCGTCCATGC-3′. The PCR product was ligated into the vector pPZP211-*CER6* previously constructed by our group (Kinoshita et al., 2011). The resulting plasmid was introduced into *Agrobacterium* *tumefaciens* (GV3101), which was next transferred to plants using the floral dip method (Clough and Bent, 1998). Transgenic plants were selected on Murashige–Skoog agar plates containing the relevant antibiotic. F_4_ homozygous plants were used in all experiments.

## Results

### ABA induces expression of ABA-responsive genes in both the rtl1 mutant and WT plants

**Figure 1:**
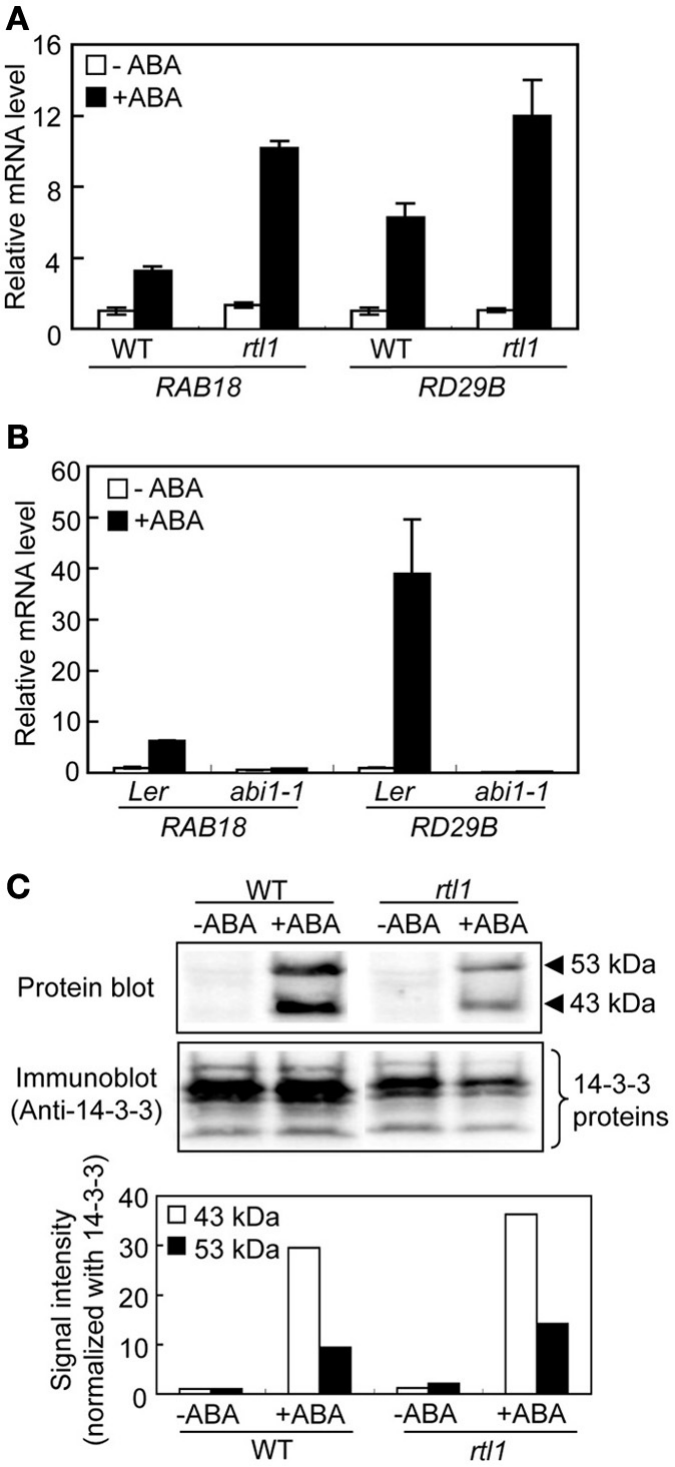
**ABA-responsive gene expression and ABA-induced phosphorylation of bHLH transcription factors (the AKSs) in guard cells from the *rtl1* mutant. (A)** The expression levels of ABA-responsive genes, *RAB18* and *RD29B*, in GCPs from WT and *rtl1* mutant plants, measured using real-time quantitative PCR. Transcript levels were normalized to that of *TUB2*. GCPs were incubated in the presence or absence of 20 μM ABA for 1 h. Data represent the means of three measurements ± standard deviations (SDs). **(B)** Expression of ABA-responsive genes in GCPs from *abi1-1* and L*er* plants. The experimental conditions were as described in **(A)** above. **(C)** ABA-induced protein phosphorylation in GCPs from WT and *rtl1* plants. Phosphorylation of 43- and 53-kDa proteins was detected via protein blot analysis using GST-fused 14-3-3 protein as a probe (“Protein blot”), and endogenous 14-3-3 proteins were detected by immunoblotting using an anti-14-3-3 protein antibody (“Anti-14-3-3 protein”). GCPs were incubated in the presence or absence of 20 μM ABA for 10 min. The lower panel shows the relative phosphorylation levels of the AKSs, and the 43- and 53-kDa proteins, quantified by calculating the ratios of the signal intensities of the protein blot bands to those of immunoblot bands of 14-3-3 proteins. The data are expressed relative to the level of phosphorylation in ABA-untreated WT plants. The experiment was repeated twice on different occasions and yielded similar results.

A recent study using a novel *chlh* mutant, *rtl1*, indicated that CHLH affects ABA-induced stomatal closure, but does not serve as an ABA receptor in *A. thaliana*, and that CHLH also does not affect other ABA-mediated phenomena, including seed germination and post-germination growth (Tsuzuki et al., 2011). However, whether CHLH affects ABA-induced expression of particular genes and/or ABA-induced phosphorylation of the bHLH transcription factors, the AKSs (Takahashi et al., 2013), in guard cells remains unclear. First, we performed quantitative PCR to determine the expression levels of typical ABA-responsive genes, *RAB18* and *RD29B* (Leonhardt et al., 2004; Fujii et al., 2007), in GCPs. Incubation of WT GCPs with 20 μM ABA for 1 h induced expression of *RAB18* and *RD29B*. Similarly, the expression levels of these genes increased in GCPs from the *rtl1* mutant in response to ABA (Figure 1A). In contrast, GCPs from the *abi1-1* mutant, which is an ABA-insensitive mutant with a lesion in the PYR/PYL/RCARs-PP2Cs-SRK2/SnRK2s pathway (Ma et al., 2009; Park et al., 2009; Nishimura et al., 2010), did not show ABA-induced gene expression (Figure 1B). These results indicated that *CHLH* did not affect the expression of ABA-responsive genes. Notably, the extent of induction of ABA-responsive genes in the *rlt1* mutant was greater than seen in the WT. In accord with this, another CHLH missense mutant, *cch* (P642 to L), which shows ABA-insensitive phenotype in stomatal closure (Tsuzuki et al., 2011), also showed higher magnitude of induction of ABA-responsive genes in GCPs (Supplementary Figure S1). Further investigation will be needed to clarify greater induction of ABA-responsive genes in these mutants.

Next, we performed protein blot analysis using 14-3-3 protein as a probe to examine ABA-induced phosphorylation of AKSs in GCPs because the 14-3-3 protein specifically binds to phosphorylated AKSs (Takahashi et al., 2013). As shown in Figure 1C, ABA-induced phosphorylation of AKSs (53 and 43 kDa in size) was evident in both WT and *rtl1* GCPs. These results indicated that *CHLH* had no effect on ABA-induced phosphorylation of AKSs.

### CHLH affects ABA-induced inhibition of BL-induced phosphorylation of guard cell H^+^-ATPase

**Figure 2:**
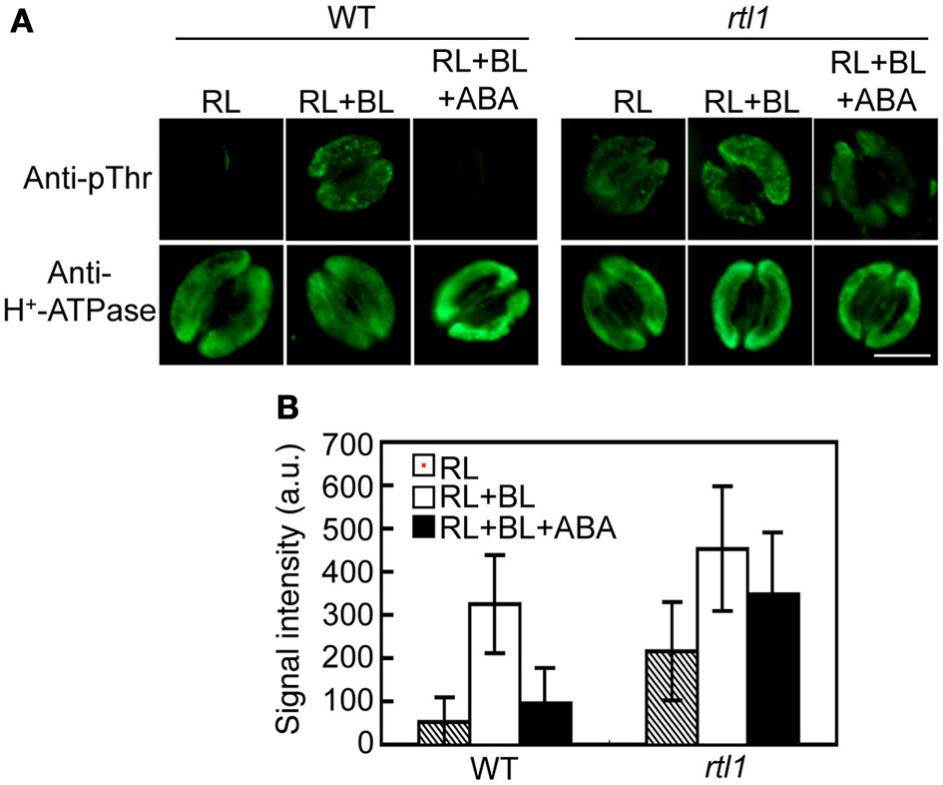
**Effect of ABA on blue light (BL)-induced H^+^-ATPase phosphorylation in stomatal guard cells of the *rtl1* mutant**. Epidermal fragments of rosette leaves from WT and *rtl1* mutant plants were illuminated with red light (RL) for 20 min and then illuminated by BL superimposed on the background RL (RL+BL) for 2.5 min. ABA (20 μM) was added to epidermal fragments 20 min before BL illumination commenced (RL+BL+ABA). **(A)** Typical images of stomata immunostained using anti-pThr and anti-H^+^-ATPase antibodies. Scale bar = 10 μm. **(B)** Fluorescent signal intensities from stomata immunostained with anti-pThr antibody. Values represent the mean ± *SD* (*n* = 30) in arbitrary units (a.u.). The experiment was repeated three times on different occasions with similar results.

Previous work indicated that ABA inhibits BL-mediated stomatal opening at least in part via induction of the dephosphorylation of guard cell H^+^-ATPase (Zhang et al., 2004; Hayashi and Kinoshita, 2011; Hayashi et al., 2011). Thus, we used an immunohistochemical method to investigate the phosphorylation status of guard cell H^+^-ATPase in the epidermis. In WT plants, guard cell H^+^-ATPase was dephosphorylated under exposure to RL, and BL superimposed on RL induced phosphorylation of the H^+^-ATPase. Furthermore, preincubation of the epidermis with 20 μM ABA completely suppressed this BL-induced phosphorylation. Moreover, inhibition of BL-induced phosphorylation of the H^+^-ATPase by ABA was severely suppressed in the *rtl1* mutant (Figures 2A,B). In addition, the mutant exhibited relatively higher phosphorylation levels of guard cell H^+^-ATPase (compared to the WT) both before and after BL illumination. The observed changes in the phosphorylation level of H^+^-ATPase were not attributable to differences in the amounts of H^+^-ATPase between strains; the treatment used had no effect on the levels of guard cell H^+^-ATPase in WT or *rtl1* plants. These results suggest that CHLH mediates not only the induction of ABA-induced stomatal closure but also the ABA inhibition of BL-induced phosphorylation of guard cell H^+^-ATPase.

### CHLH transgenic plants exhibit closed-stomata and drought tolerance phenotypes

**Figure 3:**
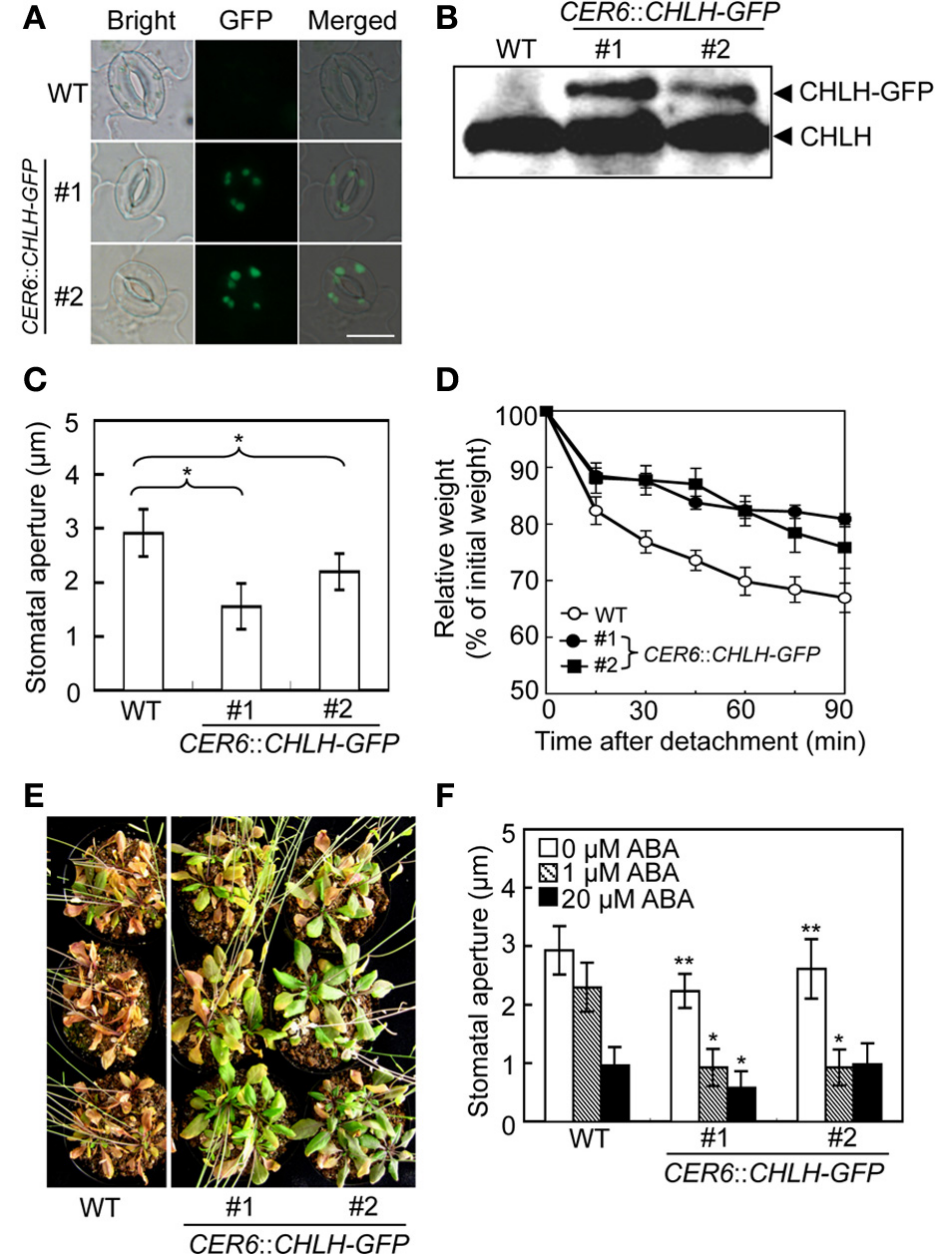
**Phenotypic analysis of *CHLH* transgenic plants**. The phenotypes of two independent *CER6::CHLH-GFP* plants were compared with that of WT plants. **(A)** Typical bright-field and GFP fluorescent images of stomata in the abaxial epidermis of rosette leaves. Scale bar = 10 μm. **(B)** Immunoblots using anti-CHLH antibody showing CHLH-GFP and endogenous CHLH in GCPs. **(C)** The stomatal aperture in 4-week-old plants exposed to light conditions at zeitgeber time (ZT) 4. Epidermal fragments were isolated from rosette leaves and apertures measured immediately. Data represent the means of 25 measurements ±SDs. Pairs for Student's *t*-test are indicated with brackets (^*^P < 0.01). **(D)** Kinetics of fresh weight changes in detached rosette leaves from 4-week-old WT plants (open circles) and *CER6::CHLH-GFP* #1 (closed circles) and #2 (closed squares). The relative weights of leaves are presented (±*SD*) as percentages of initial weights, being the weights of rosette leaves immediately after detachment from plants (*n* = 6). **(E)** Drought tolerance in WT and *CHLH-GFP* transgenic plants. Plants grown in soil for 3 weeks were subjected to drought stress by withholding water for 18 days. **(F)** Effect of ABA on the stomatal aperture of WT and *CHLH-GFP* transgenic plants. Epidermal fragments isolated from rosette leaves of 4-week-old plants exposed to light at ZT 4 were treated with ABA at the indicated concentrations for 2.5 h under BL superimposed on background red light. The stomatal apertures in the abaxial epidermis were measured microscopically. Data represent the means of 25 measurements ±*SD*s. Asterisks indicate significant differences between wild type and each *CHLH*-transgenic plants under 0 μM ABA (^*^P < 0.05; Student's *t*-test). The experiment was repeated three times on different occasions with similar results.

Knockdown of *CHLH* is reportedly associated with open-stomata and ABA-insensitive phenotypes of stomatal guard cells (Shen et al., 2006; Legnaioli et al., 2009; Tsuzuki et al., 2011), suggesting that overexpression of *CHLH* in guard cells may induce stomatal closure and increase sensitivity to ABA. We therefore constructed *CHLH-GFP* transgenic plants using the *CER6* promoter to drive overexpression of *CHLH-GFP* in epidermal tissue including guard cells (Hooker et al., 2002; Kinoshita et al., 2011). As shown in Figure 3A, GFP-derived fluorescence was observed in guard cells from *CHLH-GFP* transgenic plants. Immunoblotting of epidermal fragments revealed that CHLH-GFP was specifically detected in *CHLH-GFP* transgenic plants, in addition to endogenous CHLH (Figure 3B). Note that the epidermal fragments used in immunoblotting analysis contained not only epidermal and guard cells but also mesophyll cells attached to the epidermis (Supplementary Figure S2). Therefore, the signal from endogenous CHLH likely emanated from mesophyll cells in addition to epidermal tissue. According to the available expression database, Arabidopsis eFP Browser, expression level of *CER6*, which is used for expression of *CHLH-GFP*, is higher than that of the endogenous *CHLH* in guard cells. Thus, amount of CHLH-GFP may be higher than endogenous CHLH in guard cells. In contrast, CHLH-GFP was expressed only in epidermal tissue because *CHLH-GFP* expression was driven by the epidermis-specific *CER6* promoter. The signal intensity of GFP from *CHLH-GFP* showed that the recombinant protein was expressed principally in guard cells of the epidermis (Figure 3A).

We next measured stomatal aperture under conditions of light exposure. The stomata of *CHLH-GFP* transgenic plants were notably more closed than those of WT plants (Figure 3C). Moreover, leaves detached from *CHLH-GFP* transgenic plants lost weight only slowly under light-exposure conditions (Figure 3D). The average weight of rosette leaves detached from WT plants fell to about 70% of their initial weight over 90 min, whereas the weights of leaves from *CHLH-GFP* transgenic plants decreased to about 80% of their initial weight. These results indicate that *CHLH-GFP* transgenic plants express the closed-stomata phenotype even under conditions of light exposure.

Next, we examined drought tolerance in *CHLH-GFP* transgenic plants. Plants were grown for 3 weeks under normal conditions and next exposed to drought stress by withholding water for 18 days. Most WT plants died, while all *CHLH-GFP* transgenic plants survived. To assess whether the enhanced drought tolerance of *CHLH-GFP* transgenic plants was attributable to an alteration in sensitivity to ABA, we measured the effect of ABA on the stomatal aperture (Figure 3F). The results showed that the stomata of *CHLH-GFP* transgenic, but not WT, plants closed in response to a low concentration (1 μM) of ABA, although a high level of ABA (20 μM) induced stomatal closure in both types of plants. It should be noted that the differences of stomatal aperture between wild type and *CHLH*-transgenic plants in Figure 3F (0 μM ABA) were smaller than that in Figure 3C, since the epidermal tissues were incubated with a buffer containing 50 mM K^+^, which facilitates stomatal opening, for 2.5 h under light in Figure 3F. However, the differences were significant. Together, the results indicate that overexpression of *CHLH* in guard cells confers drought tolerance via promotion of stomatal closure and that this is probably attributable to enhanced sensitivity of guard cells to ABA.

## Discussion

### CHLH specifically affects ABA-dependent stomatal closure but not ABA-induced gene expression in guard cells

A previous study using a novel *chlh* mutant, *rtl1*, indicated that CHLH specifically affected ABA-induced stomatal closure (Supplementary Figure S3) but not germination or post-germination growth (Tsuzuki et al., 2011). In the present study, we have provided evidence that *CHLH* has no effect on the expression of ABA-responsive genes, such as *RAB18* and *RD29B*, in guard cells. A study suggested that the expression of ABA-responsive genes is mediated by an early ABA-signaling pathway, PYR/PYL/RCARs-PP2Cs-SRK2/SnRK2s (Saavedra et al., 2010). Together, the results suggest that *CHLH* specifically affects ABA-dependent stomatal closure but not regulation of ABA-responsive genes in guard cells via the PYR/PYL/RCARs-PP2Cs-SRK2/SnRK2s pathway (Figure 1A). Recently, three bHLH transcription factors, the AKSs, which control the expression of inward-rectifying K^+^ channels of the plasma membrane (including KAT1 and AKT2), have been shown to become phosphorylated in guard cells in response to ABA, and such phosphorylation is most likely mediated by the PYR/PYL/RCARs-PP2Cs-SRK2/SnRK2s pathway (Takahashi et al., 2013). However, GCPs of the *rtl1* mutant exhibited normal ABA-dependent phosphorylation of AKSs (Figure 1C). The results thus also suggest that *CHLH* has no effect on PYR/PYL/RCARs-PP2Cs-SRK2/SnRK2s pathway-mediated ABA-dependent phosphorylation of AKSs in guard cells.

### CHLH affects the phosphorylation status of guard-cell H^+^-ATPase

We recently established an immunohistochemical method for detecting BL-induced and phototropin-mediated phosphorylation of plasma membrane H^+^-ATPase in the stomatal guard cells of *A. thaliana* (Hayashi et al., 2011). The technique allows one to measure the phosphorylation/activation status of guard cell H^+^-ATPase in the epidermis of a single rosette leaf, and thus eliminates the need to prepare GCPs from a large number of plants. Moreover, the method can be used to detect guard cell responses under more natural and stress-free conditions. Protoplast preparation stresses plants. We took advantage of these properties to examine the effect of ABA on BL-induced phosphorylation of guard cell H^+^-ATPase using ABA-insensitive mutants, including *abi1-1*, *abi2-1*, and *ost1-2*. The results indicated that an early ABA signaling pathway, the PYR/PYL/RCAR-PP2Cs-SRK2/SnRK2s pathway (Ma et al., 2009; Park et al., 2009; Nishimura et al., 2010), was involved in ABA-induced inhibition of BL-induced phosphorylation of guard cell H^+^-ATPase (Hayashi and Kinoshita, 2011; Hayashi et al., 2011). In the present study, we used an immunohistochemical method to examine the phosphorylation status of the guard cell H^+^-ATPase of the *rtl1* mutant and found that ABA-induced inhibition of BL-induced H^+^-ATPase phosphorylation was greatly suppressed in the *rtl1* mutant (Figures 2A,B). In addition, the *rtl1* mutant exhibited a higher level of phosphorylation of guard cell H^+^-ATPase prior to BL illumination than the WT. These results suggest that CHLH affects ABA-induced inhibition of the BL-induced phosphorylation of the penultimate Thr of guard cell H^+^-ATPase, and that this inhibition is mediated by both the PYR/PYL/RCARs-PP2Cs-SRK2/SnRK2s pathway and CHLH. Further investigation is needed to clarify the relationship between the PYR/PYL/RCARs-PP2Cs-SRK2/SnRK2s pathway and CHLH in this context.

### CHLH overexpression confers drought tolerance via promotion of stomatal closure

In the present study, we found evidence that *CHLH-GFP* transgenic plants in which the *CER6* promoter drives the *CHLH-GFP* exhibited a closed-stomata phenotype under light conditions (Figures 3C,D), and drought tolerance, probably via increased sensitivity to ABA (Figures 3E,F). Previously, *CHLH* overexpression driven by the *CaMV35S* promoter, which induces overexpression of *CHLH* in whole plant, was found to cause development of an ABA-hypersensitive phenotype in terms of stomatal movement and increased resistance to dehydration (Shen et al., 2006). However, previous study has not shown that *CHLH* overexpression causes drought tolerance. In addition, it is worthy of note that expression of *Vicia faba VfPIP*, a putative aquaporin gene, driven by the *CaMV35S* promoter in *A. thaliana* has been shown to improve their drought tolerance at least in part through lower transpiration phenotype, although the *VfPIP* transgenic plants had longer primary and lateral roots, and a greater number of lateral roots than the control plants (Cui et al., 2008). To our knowledge, the present result is the first report to demonstrate that overexpression of *CHLH* in guard cells confers drought tolerance via manipulation of the stomatal aperture. Our results suggest that manipulation of the stomatal aperture via overexpression of *CHLH* in guard cells may prove very useful to improve drought tolerance in plants of economic importance.

## Conflict of interest statement

The authors declare that the research was conducted in the absence of any commercial or financial relationships that could be construed as a potential conflict of interest.

## Supplementary material

Supplementary Figure S1 **ABA-responsive gene expression in guard cells from *cch***. Expression level of ABA-responsible genes, *RAB18* and *RD29B* in GCPs from *cch* and wild-type (Col-0) plants determined by real-time quantitative PCR. Transcript abundance of each gene was normalized to *TUB2*. GCPs were incubated in the presence or absence of 20 μM ABA for 1 h. Data represent means of three technical measurements with the standard deviation (SD).

Supplementary Figure S2 **Typical epidermal fragments used in the present study**. Epidermal fragments were obtained by blending rosette leaves and subsequent collection of material on 58-μm-pore-sized nylon mesh as described in the *Materials and Methods*. Scale bar = 50 μm.

Supplementary Figure S3 **Effect of ABA on stomatal aperture of the *rtl1* mutant**. Epidermal fragments isolated from dark-adapted *rtl1* and WT plants were illuminated with BL superimposed on the background RL (RL+BL) for 2.5 h. ABA (20 μM) was added to epidermal fragments 20 min before BL illumination commenced (RL+BL+ABA). As a control, the epidermal fragments were incubated in the dark for 2.5 h (Dk). The stomatal apertures in the abaxial epidermis was measured microscopically. The data represent the means of 25 measurements ± SDs. The experiment was repeated three times on different occasions with similar results.
