# Supplementary figures and images for: Overexpression of the Mg-chelatase H subunit in guard cells confers drought tolerance via promotion of stomatal closure in Arabidopsis thaliana
Source: Front Plant Sci. 2013 Oct 30;4:440. doi: 10.3389/fpls.2013.00440 (PMC3812566; doi:10.3389/fpls.2013.00440)

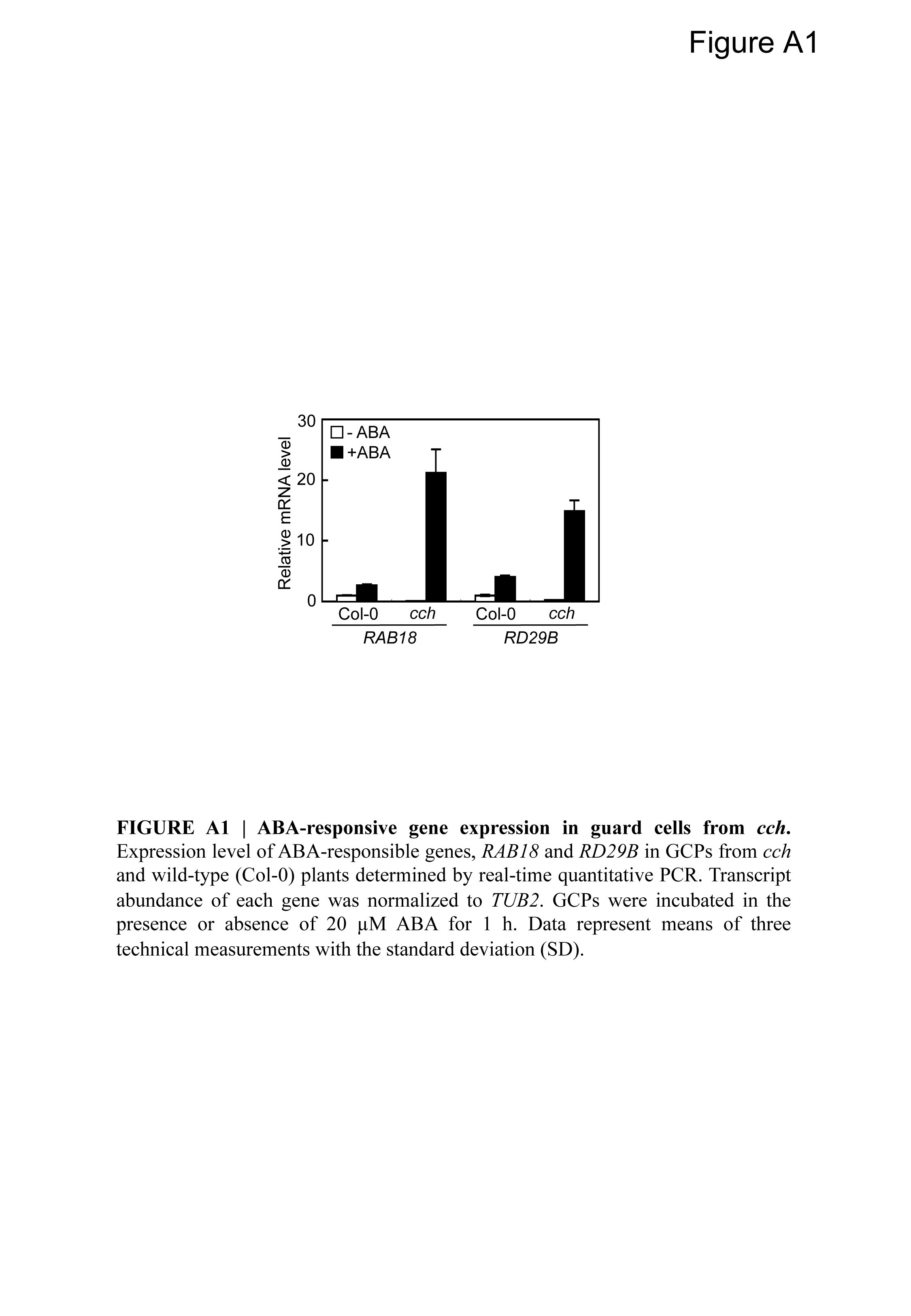

Supplement: Supplementary Figure S1 — ABA-responsive gene expression in guard cells from cch. Expression level of ABA-responsible genes, RAB18 and RD29B in GCPs from cch and wild-type (Col-0) plants determined by real-time quantitative PCR. Transcript abundance of each gene was normalized to TUB2. GCPs were incubated in the presence or absence of 20 μM ABA for 1 h. Data represent means of three technical measurements with the standard deviation (SD). [file Presentation1.ZIP › 65554_Kinoshita_Figure_A1.JPEG]

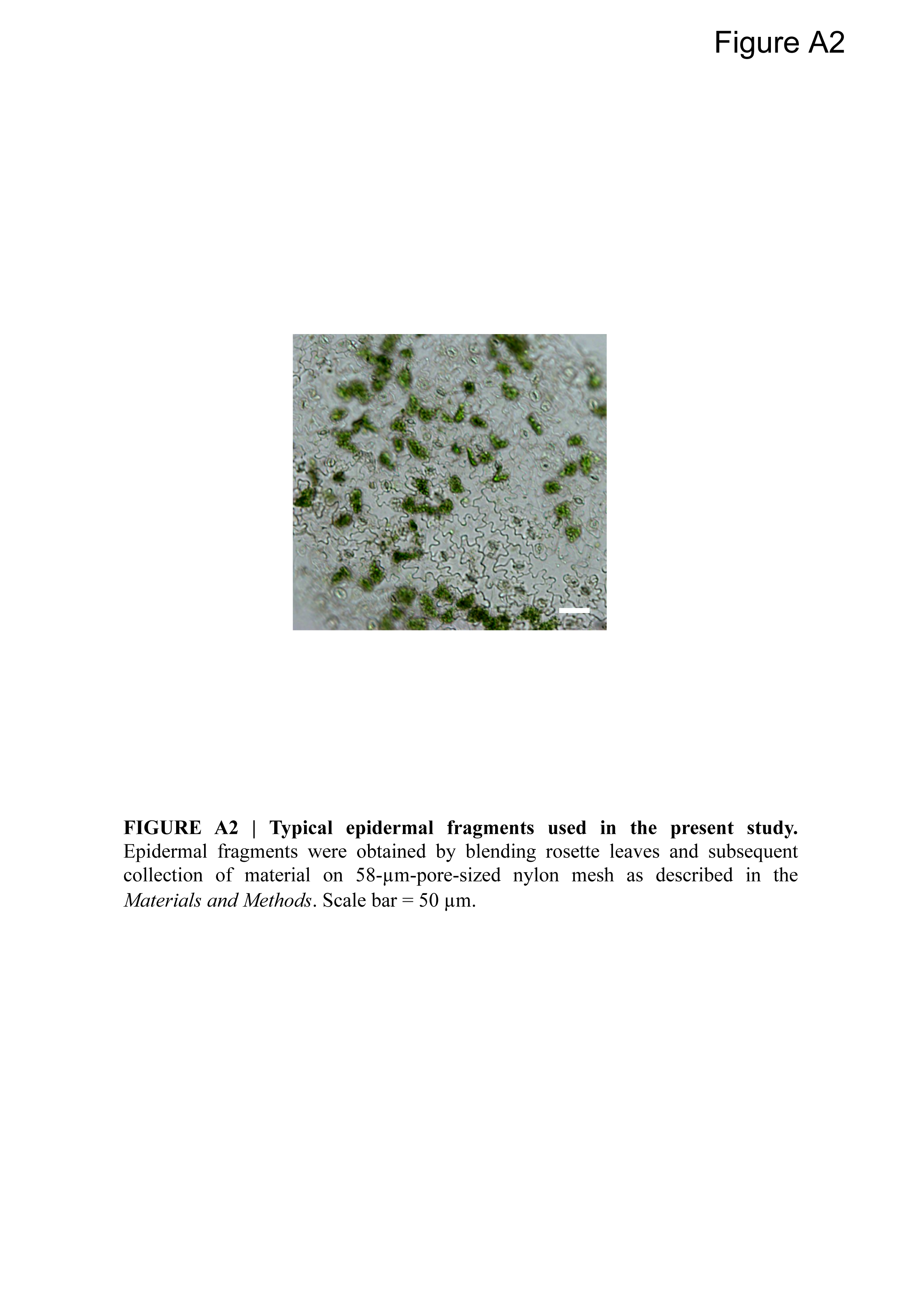

Supplement: Supplementary Figure S1 — ABA-responsive gene expression in guard cells from cch. Expression level of ABA-responsible genes, RAB18 and RD29B in GCPs from cch and wild-type (Col-0) plants determined by real-time quantitative PCR. Transcript abundance of each gene was normalized to TUB2. GCPs were incubated in the presence or absence of 20 μM ABA for 1 h. Data represent means of three technical measurements with the standard deviation (SD). [file Presentation1.ZIP › 65554_Kinoshita_Figure_A2.JPEG]

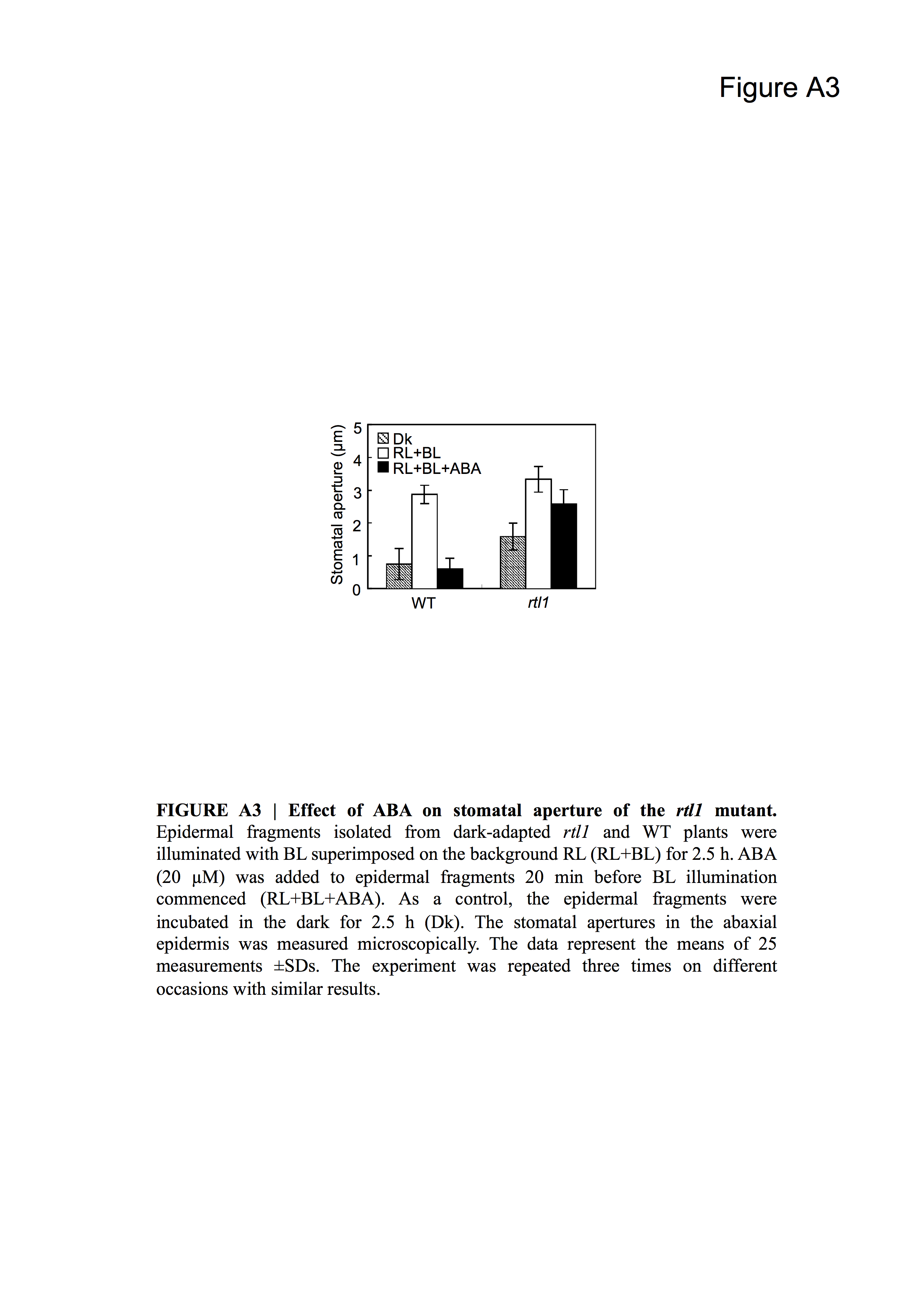

Supplement: Supplementary Figure S1 — ABA-responsive gene expression in guard cells from cch. Expression level of ABA-responsible genes, RAB18 and RD29B in GCPs from cch and wild-type (Col-0) plants determined by real-time quantitative PCR. Transcript abundance of each gene was normalized to TUB2. GCPs were incubated in the presence or absence of 20 μM ABA for 1 h. Data represent means of three technical measurements with the standard deviation (SD). [file Presentation1.ZIP › 65554_Kinoshita_Figure_A3.JPEG]
